# A portable extensional rheometer for measuring the viscoelasticity of pitcher plant and other sticky liquids in the field

**DOI:** 10.1186/s13007-015-0059-5

**Published:** 2015-03-07

**Authors:** Catherine Collett, Alia Ardron, Ulrike Bauer, Gary Chapman, Elodie Chaudan, Bart Hallmark, Lee Pratt, Maria Dolores Torres-Perez, D Ian Wilson

**Affiliations:** Department of Chemical Engineering and Biotechnology, New Museums Site, Pembroke Street, Cambridge, CB2 3RA UK; School of Biological Sciences, Life Sciences Building, 24 Tyndall Avenue, Bristol, BS8 1TQ UK; Faculty of Science, Universiti Brunei Darussalam, Tungku Link, Gadong 1410, Bandar Seri Begawan, Brunei Darussalam; ESPCI ParisTech, 10 rue Vauquelin, 75005 Paris, France; Department of Chemical Engineering, University of Santiago de Compostela, Lope Gómez de Marzoa St, Santiago de Compostela, E-15782 Spain

**Keywords:** Biological fluids, Filament, Giesekus, *Nepenthes*, Pitcher plants, Polymer solution, Polysaccharide, Rheometry

## Abstract

**Background:**

Biological fluids often have interesting and unusual physical properties to adapt them for their specific purpose. Laboratory-based rheometers can be used to characterise the viscoelastic properties of such fluids. This, however, can be challenging as samples often do not retain their natural properties in storage while conventional rheometers are fragile and expensive devices ill-suited for field measurements. We present a portable, low-cost extensional rheometer designed specifically to enable *in situ* studies of biological fluids in the field. The design of the device (named Seymour) is based on a conventional capillary break-up extensional rheometer (the Cambridge Trimaster). It works by rapidly stretching a small fluid sample between two metal pistons. A battery-operated solenoid switch triggers the pistons to move apart rapidly and a compact, robust and inexpensive, USB 3 high speed camera is used to record the thinning and break-up of the fluid filament that forms between the pistons. The complete setup runs independently of mains electricity supply and weighs approximately 1 kg. Post-processing and analysis of the recorded images to extract rheological parameters is performed using open source software.

**Results:**

The device was tested both in the laboratory and in the field, in Brunei Darussalam, using calibration fluids (silicone oil and carboxymethyl cellulose solutions) as well as *Nepenthes* pitcher plant trapping fluids as an example of a viscoelastic biological fluid. The fluid relaxation times ranged from 1 ms to over 1 s. The device gave comparable performance to the Cambridge Trimaster. Differences in fluid viscoelasticity between three species were quantified, as well as the change in viscoelasticity with storage time. This, together with marked differences between *N. rafflesiana* fluids taken from greenhouse and wild plants, confirms the need for a portable device.

**Conclusions:**

Proof of concept of the portable rheometer was demonstrated. Quantitative measurements of pitcher plant fluid viscoelasticity were made in the natural habitat for the first time. The device opens up opportunities for studying a wide range of plant fluids and secretions, under varying experimental conditions, or with changing temperatures and weather conditions.

**Electronic supplementary material:**

The online version of this article (doi:10.1186/s13007-015-0059-5) contains supplementary material, which is available to authorized users.

## Background

### Viscoelastic behaviour of biological fluids

Water has long been recognised as the essence of life, and many ubiquitous biological fluids such as cytoplasm, blood and plant sap are based on water. In contrast to pure water, aqueous (and other) biological fluids often exhibit non-Newtonian behaviour such as shear-thinning (e.g. blood [1], bronchial mucus [2], gastropod foot mucus [3] and the adhesive fluids of insects [4]). Soluble long chain polymers give rise to viscoelastic behaviour and the ability to form filaments of liquid that can stretch [5]. This impacts a broad range of biological processes from the locomotion of sperm through cervical mucus [6] to the spinning of spider silk [7] and the trapping of insects by carnivorous plants (genera *Drosera* [8], *Drosophyllum*, *Pinguicula* and *Nepenthes* [9]).

### Limitations of current rheometry methods

Accurate measurement of viscoelastic fluid properties, using extensional rheometry, is essential for understanding their contribution to the biological function. As part of living organisms, biological fluids often undergo marked changes over time [10,11], and fluid properties need to be monitored at short intervals in the natural environment in order to investigate these dynamic processes and their effects. Rigorous quantitative measurements are currently not possible in the field (laboratory devices are expensive, fragile and not readily transported), while the viscoelastic properties of many natural liquids change after sampling. Resins and latex are examples which change properties rapidly when exposed to air. Furthermore, the fluid properties depend on environmental factors such as temperature and air humidity, while the size and immobility of traditional extensional rheometers prohibits their use in climate control chambers. There is therefore a need for a portable device to study viscoelastic biological fluids *in situ* or under controlled environmental conditions.

This paper reports the development of such a device, which arose from the desire to study pitcher plant fluids *in situ* in Borneo (it was consequently named Seymour after the owner of a carnivorous plant in the movie ‘Little Shop of Horrors’). The device can be used for routine testing as well as field work. It offers the following advantages:

- It is lightweight, robust, easy to assemble and has few moving parts;
- It is constructed mainly from standard parts, which can be replaced readily, and is therefore relatively inexpensive;
- It employs small sample volumes (<10 μL), which fits the increasing demand for the miniaturisation of rheometric techniques owing to the limited availability or high cost of samples [12];
- It is easy to operate. Data can be analysed directly or remotely;
- It is suitable for testing over a broad range of temperatures and humidity levels as it fits readily into a controlled environment chamber: many studies of extensional rheology to date have been limited to standard laboratory conditions [13].

### Pitcher plant fluids

We used pitcher plant fluids as an example of a viscoelastic biological fluid in order to test the performance of the Seymour device in the laboratory and in the field. *Nepenthes* pitcher plant fluids are sticky aqueous solutions of polysaccharides [9] held in cup-shaped leaves to trap insects. Prey struggling at the fluid surface quickly cover themselves in sticky threads, much like a piece of bread is covered by molten cheese in a fondue. The plant subsequently digests the drowned insects to release and absorb mineral nutrients. Bauer *et al*. [14] compared different pitcher plant species in the field using a crude measure of extensional viscosity, namely the length of filament that could be formed by stretching the fluid between two fingers. In addition to marked differences in apparent fluid viscoelasticity between species they found strong variation between individual plants. Observations on greenhouse plants of *N. rafflesiana* further suggest that greenhouse cultivation affects fluid viscoelasticity negatively (Bauer, unpublished). It is not clear whether this is a result of suboptimal growth conditions leading to reduced polysaccharide production by the plant, or due to dilution of the pitcher fluid when the plants are watered.

Erni *et al*. [8] studied the extensional behaviour of *Drosera* mucilage using a microscope-based CaBER™ device and reported phenomena such as beads-on-string formation associated with viscoelastic fluids, with a relaxation time of *circa* 0.33 s. Gaume and Forterre [9] measured the filament thinning of *N. rafflesiana* fluid with a simple rod arrangement and reported viscoelastic behaviour with a relaxation time of *circa* 1 s. This relaxation time was longer than the period in which insect prey were observed to flex limbs and was therefore interpreted as an adaptation for prey capture. Gaume and Forterre collected fluid samples in the field [9] but it is not clear if the viscoelastic testing was performed on location. Their method also required a pool of liquid to withdraw the rod from, which is not feasible for smaller sample volumes such as the mucilage droplets of *Drosera*, *Drosophyllum* and *Pinguicula*.

### Extensional rheometry

Extensional rheology is the study of the deformation of material under conditions of pure strain; in this paper the exact form of the strain is the Hencky strain. The more commonly studied case is that of deformation under pure shear, where a sample is placed between two surfaces which move past one another, keeping the separating distance constant (or where the fluid is allowed to flow along a pipe so that the fluid shears against itself). Extensional rheometry requires the material to be stretched, and generating a reproducible stretching requires a precise mechanical action. Unlike Newtonian liquids, the extensional behaviour of viscoelastic fluids cannot be estimated reliably from measurements of shear rheology so direct measurements are necessary.

There are several methods for measuring extensional rheology [5]. Filament stretching is now a routine method in the laboratory, using devices such as the FiSER™ (Cambridge Polymer Group, Boston), CaBER™ [15] (Cambridge Polymer Group, Boston and Haake), and the Cambridge Trimaster [16] to measure the necking of an extensionally-strained fluid filament as a function of time. The evolution of the filament diameter can be used to identify the nature of the fluid (*e.g*. Newtonian, viscoelastic *etc.*, see next paragraph) and, given the surface tension of the fluid, to calculate the extensional viscosity. Viscoelastic fluids are characterised by viscous and elastic contributions: an important parameter in the latter is the relaxation time, *λ*, which is simply speaking the time needed for the polymer molecules to adjust to a change in strain.

**Figure 1:**
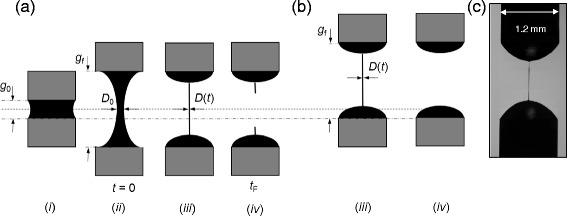
**Schematic of CaBER™** **operation. (** **a** **)** standard mode, platens move equal distance apart. Initial gap *g*_0_, final gap *g*_f_. **(** **b** **)** Seymour operation, only upper platen moves. **(** **c** **)** Example of *N. maxima* filament shortly before breakup, *g*_0_ = 0.4 mm. Time set to zero when platens stop moving, (*ii*); filament width measured (*iii*) until break up at *t*_F_, (*iv*).

A review of filament stretching rheometers can be found in Galindo-Rosales *et al*. [17]. A sample of liquid is placed between two platens (*i.e*. the ends of two rods), and the platens moved apart either (*i*) at controlled separating speed, imparting a known strain rate, or (*ii*) rapidly, causing the formation of a filament which thins under capillary action. The latter mode, shown in Figure 1(a), known as CaBER™ (capillary break-up extensional rheometry [18]) mode, is used here. A high speed camera and image analysis software monitors the filament diameter at its mid-point, *D*, over time, *t*. The filament will break at the mid-point under pure extension. Gravity has negligible effect with the devices employed here, as the dimensions give Bond numbers « 1 [9]; this is discussed after Equation (7).

The evolution of *D* with respect to its initial value, *D*_0_, has been determined for various constitutive fluid models, and those considered here are:

- Newtonian liquid, with constant shear viscosity, *η*_0_, and surface tension, *α* [19]. A correction factor, *X*, has been included to account for the non-cylindrical nature of the fluid filament: *X* = 1 for ideal, cylindrical filaments; *X* = 0.7127 for non-inertial, smooth, filaments. More information on this correction, and on the systematic differences in Newtonian viscosity calculated by extensional and shear measurements, can be found in McKinley and Tripathi [15] and Liang and Mackley [20].
  1 $$ \frac{D}{D_0}=1\hbox{-} \frac{\left(2X-1\right)\alpha t}{3{\eta}_0{D}_0} $$
- The Upper Convected Maxwell (UCM) model, representing the simplest viscoelastic fluid model [19] for negligible viscosity with relaxation time, *λ*_UCM_;
  2 $$ \frac{D}{D_0}= \exp \left(\frac{-t}{3{\lambda}_{UCM}}\right) $$
- The Giesekus model for viscoelastic solutions [21], which includes a relaxation time, *λ*_G_, and a polymer interaction term, the mobility parameter, *a*, gives the following implicit relationship for *D*/*D*_0_ [22];
  3 $$ \left(4a-3\right) \ln \left(\frac{D/{D}_0+2\alpha {\lambda}_Ga/{\eta}_0{D}_0}{1+2\alpha {\lambda}_Ga/{\eta}_0{D}_0}\right)-\frac{2{D}_0{\eta}_0}{\alpha {\lambda}_G}\left(D/{D}_0-1\right)=\frac{t}{\lambda_G} $$
  Numerical fitting techniques are needed to extract the Giesekus model parameters from filament thinning data sets.

As the filament thins it will break due to capillary instability. The time where this occurs will depend on the nature of the filament but an estimate of the filament break-up time, *t*_F_, can be obtained by setting *D*/*D*_0_ = 0. For a Newtonian fluid, Equation (1) gives

4 $$ {t}_F\approx \frac{3{\eta}_0{D}_0}{\alpha \left(2X-1\right)} $$

### The Seymour concept

In the Trimaster (and other devices), the platens move apart so that the filament midpoint remains at the same location, greatly reducing the computational effort in analysing images but requiring delicate mechanical action (Figure 1(a)). In contrast, the Seymour only moves one platen using a standard solenoid switch (Figure 1(b)). This improves the robustness of the device (fewer moving parts) and reduces the cost demonstrably. The major cost component is the digital camera: the device for fieldwork reported here cost approximately €2000 in June 2014. Moving only one platen also makes setting the filament size simpler. Due to the increasing resolution of modern cameras and processing power of laptop computers, the filament midpoint can be easily located by an image analysis code.

### Aims and scope of the present study

The portable rheometer (Seymour) and its use are described in detail. We performed a series of experiments in order to answer the following questions: (*i*) Does the Seymour device produce comparable results to the conventional laboratory rheometer Trimaster Mk II, for both standardised synthetic liquids and natural pitcher plant fluids? (*ii*) Does the device yield reliable measurements in the field? (*iii*) Are there quantifiable differences between the fluids of *Nepenthes* pitcher plants sampled in greenhouses and in the field? The last two questions were investigated in order to quantify the advantages of a device that allows for on-site measurements of fluid viscoelasticity.

## Results and discussion

### Benchmarking studies

**Figure 2:**
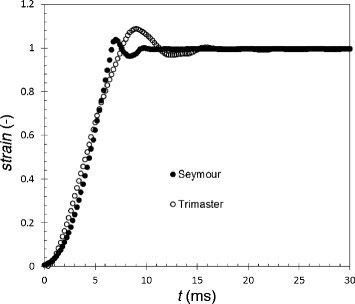
**Comparison of linear (Hencky) strain between portable (Seymour) and laboratory (Trimaster) devices.** The initial gap, *g*_0_, was 0.7 mm and reaches a final gap of 1.9 mm. Both devices produced similar separation speed. Seymour produced less overshoot and higher damping than the Trimaster.

CaBER™ testing requires a rapid initial separation of the platens in order to generate a filament, and negligible movement thereafter. Figure 2 compares the gap separations, expressed in terms of the Hencky strain, measured for the Seymour and the Trimaster for identical stretching settings. The separation speed for both devices is similar, at approximately 75 mm s^−1^. Neither device imparts a constant strain rate during the separation stage, while the Seymour produces less overshoot and faster damping than the belt-driven Trimaster. Filament diameter measurements could therefore be collected after 10 ms on the Seymour (17 ms on the Trimaster). This sets a lower limit on the viscosity of liquids that can be measured as the filament stretching should be measured after the platens have stopped moving. Equation (1) predicts that low viscosity liquids will approach filament breakup (*D*/*D*_0_ approaching zero) at short times. For example, a Newtonian 20 mPa s silicone oil exhibited filament breakup within the 10 ms initial separation period.

**Figure 3:**
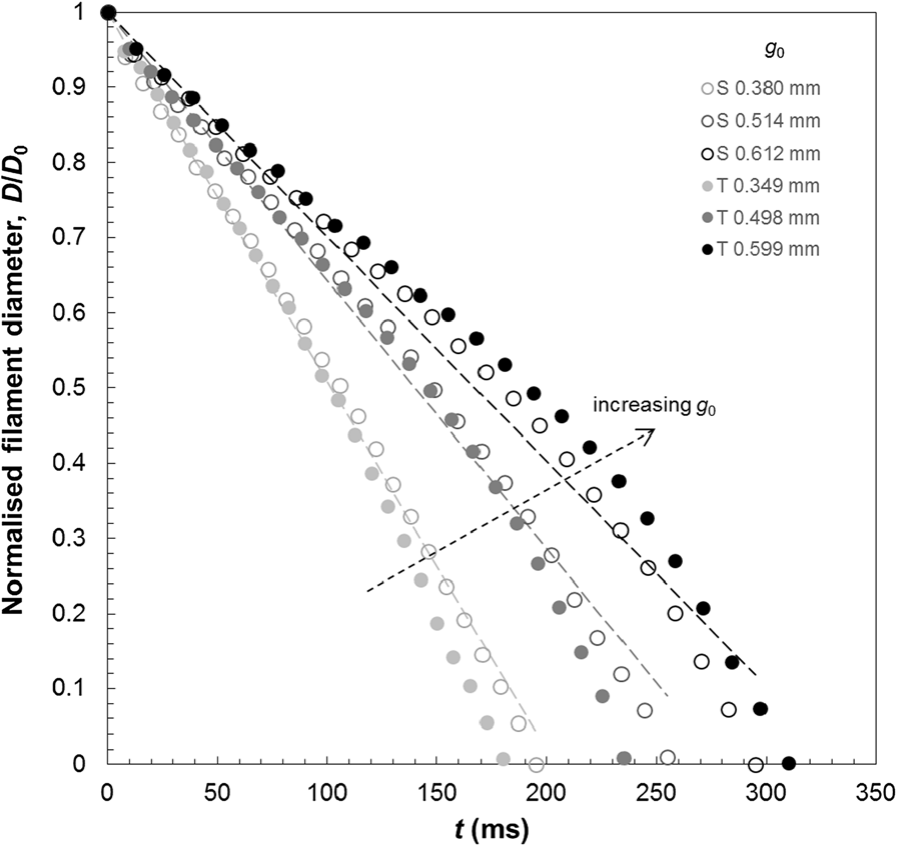
**Comparison of filament thinning behaviour for silicone oil.** Filament diameters decreased linearly as expected for a Newtonian liquid. Smaller initial gap sizes yielded a better linear fit. Data sets obtained with Seymour (S, open symbols) and Trimaster (T, solid symbols) devices showed strong agreement. Data are decimated for clarity.

Figure 3 shows the evolution of filament diameter for the Newtonian silicone oil obtained with the Trimaster and Seymour devices; both series exhibit an essentially linear decrease, as predicted by Equation (1). The dashed lines in this Figure represent the best fit of Equation (1) to the Seymour device’s experimental data using viscosity as an adjustable parameter: for this fitting, *X* = 1 and *α* = 0.0159 N m^−1^. The range of reported surface tension values for silicone oil in the literature lie between 0.0159 N m^−1^ and 0.0213 N m^−1^ [23]. Viscosity values of 2.37 Pa s, 2.71 Pa s and 3.1 Pa s were calculated for initial gap sizes of 0.380 mm, 0.514 mm and 0.612 mm respectively; it is notable that the accuracy of the fit of Equation 1 to these data sets decreased as a function of increasing gap size, with *R*^2^ values of 0.997, 0.989 and 0.982 respectively. The mean value of the viscosity found by these extensional measurements was 2.75 Pa s, some 16% higher than the reported value of 2.37 Pa s. The measurements suggest that smaller initial gap sizes are preferable in order to obtain accurate measurements. All gap sizes used were smaller than the capillary length, ${l}_{cap}=\sqrt{\sigma {/}_{\rho g}}$, where *ρ* is the fluid density, as recommended by other researchers in this area [24].

**Figure 4:**
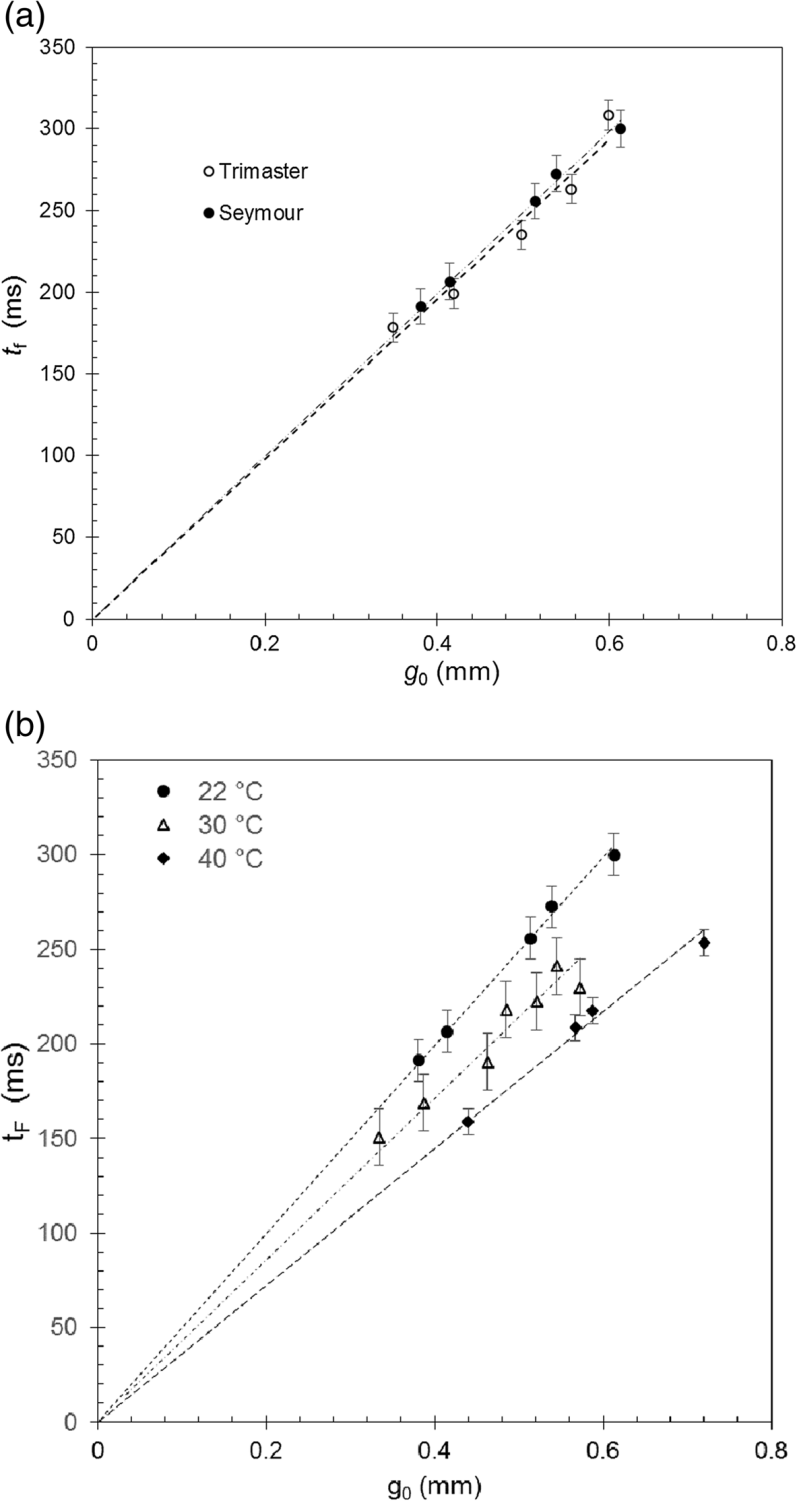
**Filament break-up times for silicone oil (Newtonian fluid). (** **a** **)** Measurements obtained with Trimaster Mk II and Seymour at 22°C show excellent agreement. **(** **b** **)** Higher temperatures lead to shorter break-up times (data obtained with Seymour device).

The gradients and filament breakup time, *t*_F_, increase with initial filament diameter, *D*_0_, as predicted by Equations (1) and (4), respectively. Figure 4(a) shows excellent agreement between the *t*_F_ values measured on the two devices at 22°C. The relationship between *t*_F_ and *g*_0_ also exhibits the trend expected for a Newtonian fluid (Equation (4)), as *D*_0_ is expected to vary with *g*_0_.

Figure 4(b) presents results obtained using the Seymour device at higher temperatures, spanning the range anticipated for field studies (up to 40°C). Filament evolution plots were linear, as in Figure 3, and *t*_F_ decreases at higher temperature. This is consistent with Equation (4), as *η*_0_ decreases with temperature.

**Figure 5:**
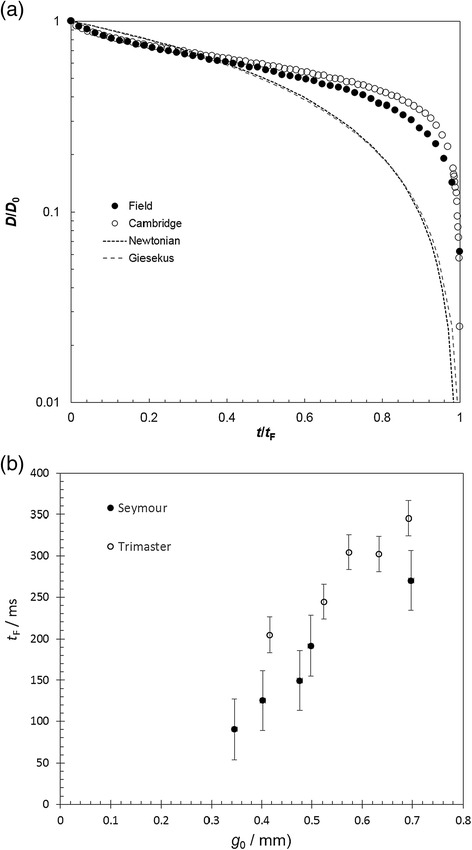
**CMC solution filament thinning behaviour showing complex behaviour. (** **a** **)** Seymour testing, alongside best fit lines for the Newtonian and Giesekus models. The data are plotted against dimensionless time, *t*/*t*_F_. Laboratory and field measurements largely agree. **(** **b** **)** Filament break-up times increased linearly with initial gap size. Seymour yielded consistently shorter break-up times than the Trimaster.

Figure 5(a) shows the results obtained for the CMC solution, which is known to be viscoelastic, measured on the Seymour in the laboratory in Cambridge and in the field in Borneo in summer 2014. The filament evolution is presented a function of dimensionless time. The use of dimensionless time accounts for the difference in temperature between the tests in Cambridge and those done in the field; additional insight into this superposition is given by Torres and co-workers [22]. Tests on the Trimaster gave similar results. There is a noticeably sharp transition to filament breakup at *t*/*t*_F_ > 0.8, which is not predicted by any of the simple constitutive models (Equations (1)-(3)). The effect of initial gap size on filament break up time is shown in Figure 5(b). The Seymour *t*_f_ values tended to be shorter than those obtained with the Trimaster. This difference may be related to the protocols: it took longer to load the Trimaster and for the final gap to stabilise, and water evaporation would increase the viscosity and thus filament breakup time. CMC solutions represent complex fluids [25] and these results confirm that the Seymour gives qualitatively similar results to the Trimaster.

The above results constitute proof-of-concept of the portable extensional rheometer (Seymour). The data from this device showed good agreement with those obtained with a precision unit, the Cambridge Trimaster Mk II. This was the case for both Newtonian (silicone oil) and complex biopolymer solutions (CMC). Moreover, the Seymour unit is small enough to fit into a climate-controlled chamber, allowing the effects of temperature and relative air humidity to be studied. The Seymour functions satisfactorily at temperatures up to 40°C, which is essential for field studies in the tropics as well as for medical studies under physiological temperatures. Humidity levels were not investigated as part of this study, but the limit in this regard in field tests is likely to be set by the camera and laptop computer. This broad operational range, together with the small size and weight of the system, renders the Seymour highly suitable for field studies.

### Application to biological (pitcher plant) fluids

Pitcher plant fluids taken from individual *N. rafflesiana*, *N. eymae*, and *N. maxima* obtained from botanical gardens (*i.e*. greenhouse plants) were tested within one day and periodically thereafter over a period of two weeks. The fresh fluids formed filaments which remained intact for some time (an example for *N. maxima* in shown in Figure 1(c)), while the *N. rafflesiana* fluid was not very viscous and the filament often broke before the platens finished moving. We only report data obtained with Seymour here as the Trimaster yielded similar results. Some samples exhibited the formation of satellite droplets, known as ‘beads on a string’ (BOAS). The presence of viscoelasticity is a pre-requisite for the formation of BOAS within fluid samples [26] and an example of this behaviour is evident in the field test on *N. rafflesiana* in the Additional file 1: video.

**Figure 6:**
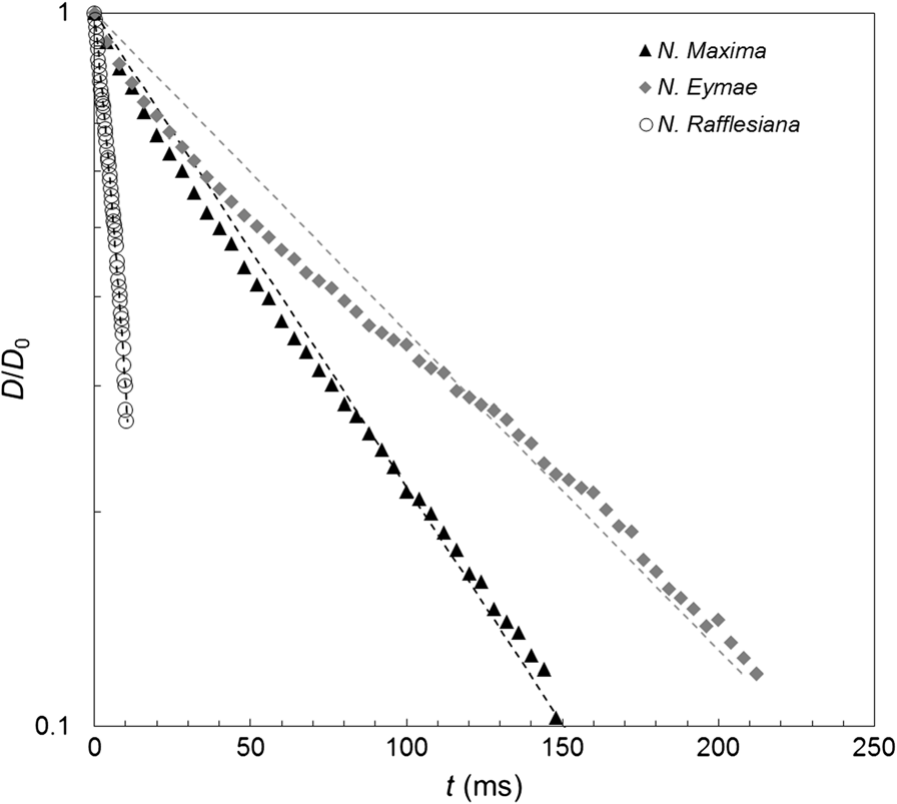
**Filament evolution profiles for fresh greenhouse-sampled of pitcher fluids.** *N. eymae* (diamonds) and *N. maxima* (triangles) both showed clearly viscoelastic behaviour while the *N. rafflesiana* filament broke before the pistons finished separating. The UCM model (dashed lines) provided the best fit for all three samples; λ_UCM_ values given in Table 1. Experimental data have been decimated for clarity.

The regression coefficients quantifying the fit of Equations (1), (2) and (3) to filament thinning profiles (plots of *D*/*D*_0_ or ln(*D*/*D*_0_) against *t*) are given in Table 1. The plots in Figure 6, of ln(*D*/*D*_0_) against time, suggested by Equation (2), show an approximately linear trend for all three fluids. This indicates that the viscoelasticity is adequately described by the simple, single parameter UCM model. The values of the relaxation time, *λ*_UCM_, extracted from model fitting are reported in Table 1. The *N. rafflesiana* value, of 3 ms, is small and could not be measured reliably with either of the Trimaster or Seymour devices. The *N. eymae*, and *N. maxima* values are more than an order of magnitude smaller than relaxation times of ~ 1 s reported by Gaume and Forterre for *N. rafflesiana* [9], confirming the desirability to perform tests in the field if possible.

**Table 1:** **Parameter estimates and goodness of fit for different fluid models for pitcher fluids from three** ***Nepenthes*** **species (samples obtained from greenhouse plants, measurements performed with Seymour)**

**Species**	**Equation (** 1 **)**		**Equation (** 2 **)**		**Equation (** 3 **)**			
	***η***_0_	***R*** ^2^	***λ***_UCM_	***R*** ^2^	***η***_0_	***a***	***λ***_G_	***R*** ^2^
	**(Pa s)**		**(ms)**		**(Pa s)**	**(−)**	**(ms)**	
*N. rafflesiana*	3.47	0.964	2.95	0.994	1.97	0	1.79	0.995
*N. eymae*	26.7	0.503	29.8	0.958	0.0644	0	32.4	0.979
*N. maxima*	20.6	0.599	20.6	0.991	0.0491	0	21.7	0.994

### Effect of sample storage on pitcher plant viscoelasticity

It has been observed that pitcher plant fluids stored for over one month lose their stickiness (Bauer, unpublished), which is an indicator of a reduction in viscoelasticity. This was reproduced in laboratory studies on (greenhouse-sourced) fluid samples of *N. maxima* and *N. eymae.* Fluids were found to lose their viscoelastic properties when stored at ambient temperatures over 2–4 weeks.

**Figure 7:**
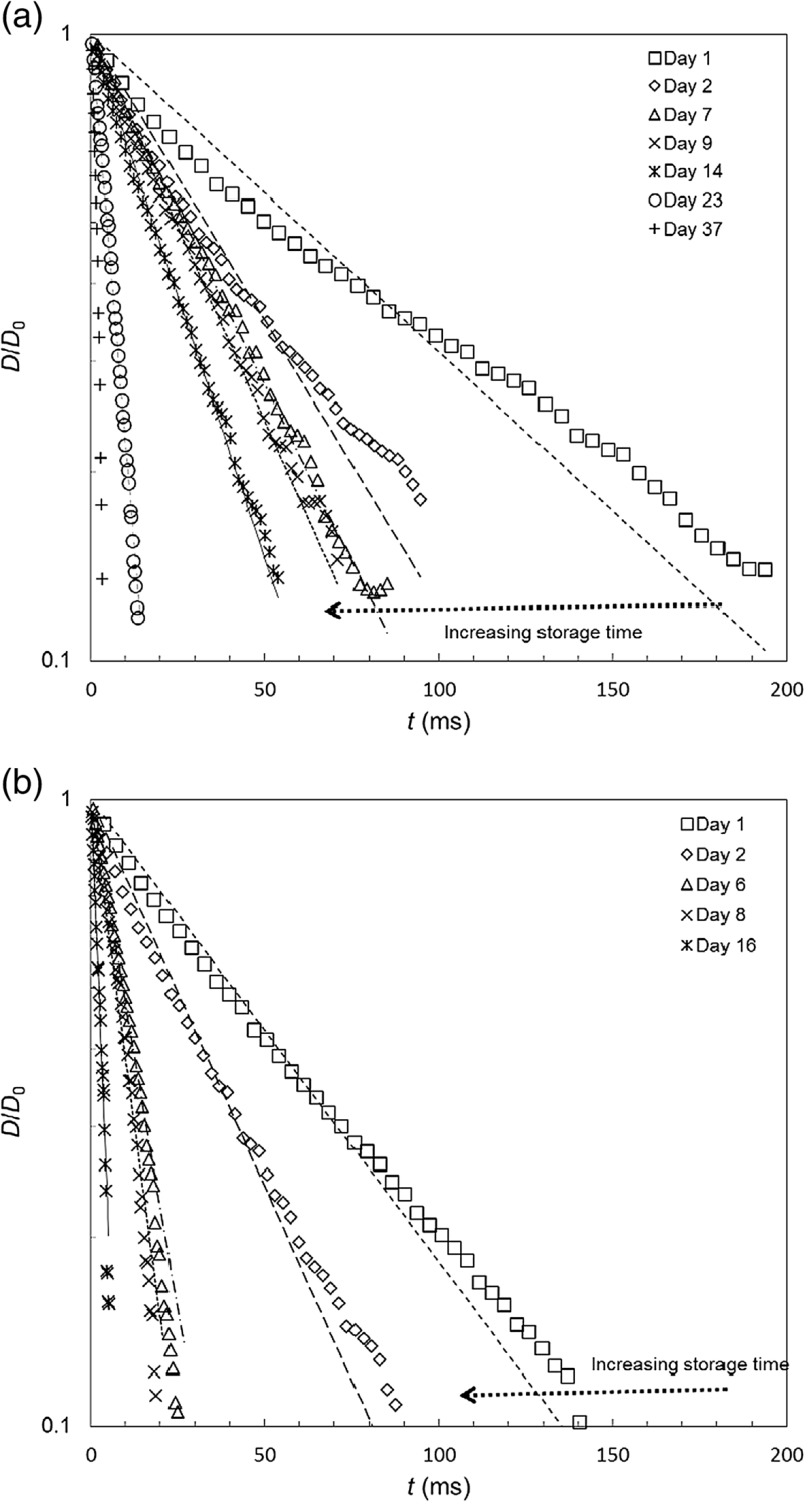
**Effect of storage time on filament thinning behaviour.** The viscoelasticity of **(** **a** **)** *N. eymae* and **(** **b** **)** *N. maxima* pitcher fluid decreased markedly with storage time. Symbols show experimental data, decimated for clarity. Lines show the fit to the UCM model, equation (2), with *λ*_UCM_ values in Table 2.

The samples were stored in sealed containers at room temperature and small aliquots were extracted for testing on the Seymour device at different times over two weeks and a month. The testing period was longer for *N. eymae* as the initial *t*_f_ value was larger and quantitative data could be obtained over a month. Figure 7 shows noticeable differences in viscoelasticity with storage time. These data were fitted to all three expressions to quantify the change in viscoelastic behaviour. The *R*^2^ values are reported in Additional file 2: Table S1. The plots show an approximately linear decrease in ln(*D*/*D*_0_) with time, indicating that the viscoelasticity is adequately described by the simple, single parameter, UCM expression and this model provided a better description for most cases for both fluids across the sample sets. The Giesekus model gave comparable *R*^2^ values but the low (sometimes zero) magnitude of *η*_o_ cast doubt on the validity of the results. The quality of fit of the Newtonian model for *N. eymae* improved considerably with storage time. This trend indicates that the fluids are losing their viscoelastic properties with time when stored at ambient temperature.

Figure 7 also shows a noticeable reduction in *t*_f_ with storage time, which is again consistent with the fluids changing from viscoelastic to Newtonian behaviour. The relaxation times extracted from model fitting are reported in Table 2 and also decrease over the storage period, by over an order of magnitude. This will give rise to predominantly Newtonian behaviour, which can be illustrated using the result for the Giesekus fluid in Equation (3). If the product of the terms is small compared to the non-dimensional filament diameter, *D*/*D*_0_, then it can be shown that

5 $$ \left(4a-3\right){\lambda}_G \ln \left(D/{D}_0\right)+\frac{2{\eta}_0}{\alpha}\left({D}_0-D\right)=t $$

**Table 2:** **Effect of storage at room temperature on the UCM relaxation time for two greenhouse-sourced pitcher plant fluids**

**Species**	**Storage time (day)**	***λ***_UCM_ **(ms)**	***R*** ^2^ **(−)**
*N. eymae*	1	28.5	0.957
	2	15.8	0.975
	7	12.9	0.997
	9	11.7	0.994
	14	8.64	0.996
	23	2.21	0.998
	37	0.73	0.950
*N. maxima*	1	19.6	0.991
	2	11.7	0.986
	6	4.25	0.991
	8	3.49	0.979
	16	1.08	0.989

Values of *λ*_UCM_ obtained by fitting Equation (2) to the *N. eymae* and *N. maxima* data in Figure 7.

**Figure 8:**
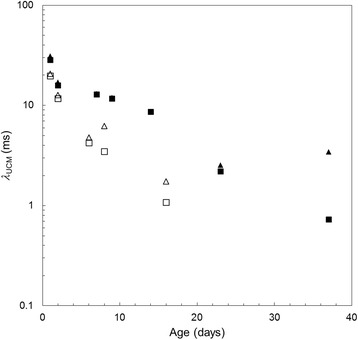
**Effect of storage time on UCM and Giesekus model relaxation time.** Relaxation time for *N. eymae* (solid symbols) and *N. maxima* (open symbols) decreased strongly with storage time. λ_G_ – triangles; λ_UCM_ – squares.

As *λ*_G_ decreases, the viscoelastic contribution (first term on the left hand side) becomes negligible and viscous behaviour dominates. Figure 8 shows how *λ*_UCM_ changes over the storage period. The almost linear trend for the *N. eymae* fluid on this log-linear plot suggests a first order decay. Elucidating this behaviour requires further work and analysis of the biopolymer components.

The above results confirmed and quantified the previously observed decay of viscoelasticity for pitcher fluids in storage. Both *N. maxima* and *N. eymae* fluids showed a tendency to become more Newtonian over the course of two to four weeks (Figure 7). The time–dependent reduction in the relaxation time of longer-stored fluid samples provided a second quantitative measure of the loss of fluid viscoelasticity (Figure 8). The two fluids considered here were sampled from newly opened pitchers so the decay is unlikely to be caused by environmental factors or by the interaction with captured prey or pitcher-colonising infauna organisms. Some insight into this behaviour was provided by storing a sample under chilled conditions, at 4°C. An aliquot was withdrawn and allowed to warm to room temperature for testing. Chilling effectively halted the change in viscoelasticity, as shown for *N. maxima* in Additional file 3: Figure S1, which may be related to inactivation of enzymes: this is the subject of ongoing work. The marked change in fluid viscoelasticity observed over a comparably short period of time further highlights the need for a portable device to measure fluid samples in the field, immediately after collecting them.

### Application in the field (Borneo 2014)

The Seymour device was tested in Borneo in summer 2014 where we used it successfully to measure field-collected pitcher plant fluids as well as the above mentioned test liquids (silicon oil, CMC). A preliminary analysis of 11 newly-opened pitchers of *N. rafflesiana* indicates that extensional thinning of the fluid is best modelled by Equation (3), indicating a Giesekus response with *R*^2^ values being essentially the same as, or higher than, those obtained using Equation (2). The model parameters and the associated *R*^2^ values are reported in Additional file 4: Table S2.

The viscoelastic properties of the *N. rafflesiana* trap fluids showed pronounced individual variation with relaxation times spanning an order of magnitude from 85 ms to 1.2 s (mean ± S.E. = 551 ± 109 ms). The upper limit of this range is comparable to the value of 1 s reported by Gaume and Forterre [2]. The mean viscosity of 25.3 ± 4.8 Pa s was similarly variable.

**Figure 9:**
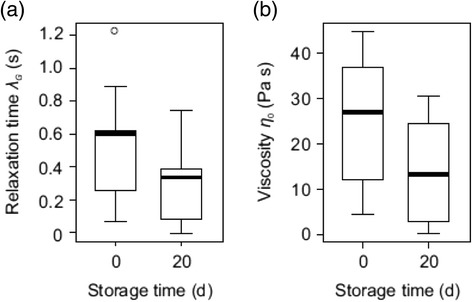
**Effect of storage at room temperature (25-30°C) on** ***N. rafflesiana*** **pitcher fluid.** Relaxation time **(** **a** **)** and viscosity **(** **b** **)** of 9 field-collected fluid samples from freshly opened *N. rafflesiana* pitchers decreased significantly (*P* < 0.5 for both; see text for statistics and Additional file 4: Tables S2 and Additional file 5: Table S3 for raw data) over a 20-day storage period at room temperature. Bars denote medians, boxes represent the inner two quartiles and whiskers include all values within 1.5 times interquartile range.

Fluid from 9 of the 11 pitchers was stored for a period of 20 days in sterile screw-top plastic vials at room temperature (25-30°C). At the end of this 20-day period, the fluid samples were re-measured using the Seymour device to determine whether the rheological parameters had changed. The model parameters obtained by fitting to the samples after storage, and associated *R*^2^ values, are reported in Additional file 5: Table S3. The relaxation time of the samples decreased significantly over the 20-day storage period (Wilcoxon-matched-pairs test, *n* = 9, *R*^ = 4.00, *P* < 0.05; Figure 9). Likewise, the mean viscosity was found to be significantly lower after storage (Paired-samples *t* test, d.f. = 8, *t* = 2.34, *P* < 0.05; Figure 9). These results confirm that there was a measurable loss of both elasticity and viscosity over 20 days of storage time which is consistent with previous observations of the loss of fluid stickiness with time [21]. They further underline the need for a reliable method for making on-site measurements as soon as possible after sample collection, especially in remote field locations where refrigeration is not available.

**Figure 10:**
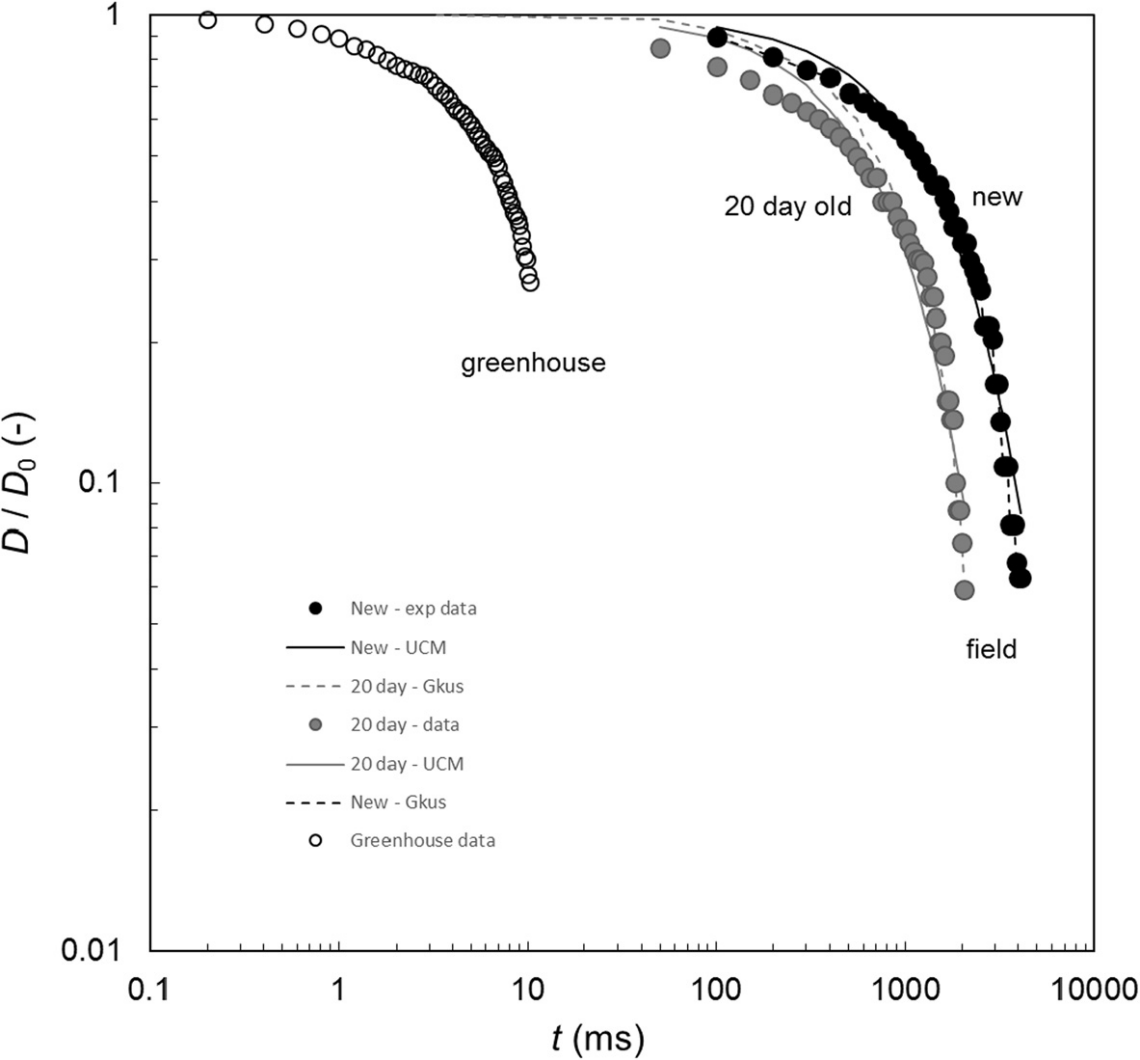
**Normalised filament profile from field and greenhouse** ***N. rafflesiana*** **pitchers.** Data show field measurements for fluid from newly-opened (black circles) and 20-day old (grey circles) *N. rafflesiana* pitchers alongside the profile for a newly-opened greenhouse plant (open circles) fluid reported in Figure 6. Data have been decimated for clarity. Data points are Seymour measurements. Solid line shows the fit to Equation (2) and dotted line shows the fit to the Giesekus fluid model, Equation (3); UCM and Giesekus parameters are those for pitcher 19 in Additional file 4: Tables S2 and Additional file 5: Table S3.

These preliminary field data also allow initial comparison to be made between typical extensional-thinning behaviour of fluid obtained from pitchers grown in the field and pitchers cultivated in greenhouses. Figure 10 shows the filament thinning profile data extracted from a newly-opened pitcher of *N. rafflesiana* having a relaxation time close to the cohort mean. Also plotted on this figure is the thinning profile extracted from the same pitcher 20 days later along with data from a newly-opened pitcher sourced from Cambridge Botanical Gardens. The fresh liquid from the plant in its native habitat was noticeably more elastic than the sample obtained from the greenhouse plant, as indicated in the Additional file 1 (video) by the strand which attached to the pipette when it was withdrawn from the pitcher. After 20 days the liquid extracted from the native pitcher, however, exhibited a noticeably reduced level of elasticity. The greenhouse sample, however, exhibited a response that shows a level of elasticity that is orders of magnitude smaller than either of the field samples. This observation illustrates the benefits of *in-situ* testing of plants in their native habitat.

### Viscoelasticity models for pitcher fluids

Also plotted in Figure 10 are the loci obtained by fitting the UCM and the Giesekus models to the data from the fresh and stored samples. The *R*^2^ values for the fresh samples are 0.984 and 0.989 for the UCM and Giesekus models, respectively. The three-parameter Giesekus model is able to provide a better fit to the experimental data as the filament rupture time is approached. The relaxation times for the freshly-opened pitcher, *λ*_G_ (620 ms) and *λ*_UCM_ (558 ms), are comparable to the the previously reported value of 1 s [9]; the relaxation times for the aged pitcher, *λ*_G_ (360 ms) and *λ*_UCM_ (284 ms), have fallen to between 50% and 60% of the newly-opened pitcher.

Figure 10 confirms the need to measure the extensional behaviour *in-situ*. There is a marked difference in the *N. rafflesiana* results for samples from greenhouse cultivated plants and those collected *in-situ* in Borneo, with the relaxation times for the latter being considerably longer, at 620 ms *vs*. 3 ms. The results for *N. rafflesiana*, *N. eymae*, and *N. maxima* from botanic gardens (Figure 6) indicate that the fluids from all three species initially possess viscoelasticity. Their viscoelastic response is adequately described by the single-parameter UCM model, which is the simplest viscoelastic fluid model available. Fluid filament rupture was observed in *N. rafflesiana* after about 10 ms, and after about 150 ms and 200 ms for *N. maxima* and *N. eymae*, respectively. The reduced viscoelasticity could be due to different growth conditions (temperature, air humidity, light levels) in the greenhouse environment, or to accidental dilution of the pitcher fluid when watering the plants.

Furthermore, whereas the simple UCM model could be used to describe the levels of viscoelasticity present in greenhouse-collected samples of all ages, the field-collected *N. rafflesiana* fluid is better described by a more complex constitutive model. The results in Figure 10 show good agreement with the single mode Giesekus fluid. Other models, such as the FENE dumbbell model, exist and may give a slightly better fit than those considered in this paper [5,27,28]; these, however, were not considered further due to the current lack of a simple analytical solution that can describe filament thinning. The benefit of this is that the factors affecting these constitutive model parameters, such as polymer nature, concentration and size, have been studied in depth for synthetic polymer solutions. Since the Seymour device now allows fluids such as pitcher plant liquids to be studied in the field (and thus free from any storage artefacts), variations between species and age may now be related to these polymer characteristics with greater confidence.

## Conclusions

A robust and reliable portable extensional rheometer (Seymour) was developed and commissioned using silicone oil and CMC solutions as test fluids. Results obtained on the Seymour device showed good agreement with those obtained with a precision unit, the Cambridge Trimaster. The Seymour unit also fitted into a climate chamber, allowing the effect of temperature and humidity on the viscoelastic behaviour of fluids to be studied.

The extensional rheology of four pitcher fluid samples sourced from greenhouses, and a further 11 samples measured in the field, were investigated. Three of the four greenhouse samples exhibited significant viscoelasticity, which could be modelled using the Upper Convected Maxwell model. The field sample exhibited stronger viscoelasticity, with relaxation times two orders of magnitudes greater, and were best described by the Giesekus model. The viscoelasticity of greenhouse- and field-collected samples alike decreased significantly during storage.

### Perspective: limitations and potential applications

The Seymour concept for a portable extensional rheometer has been demonstrated. The device was successfully employed in a field study investigating pitcher plant fluids in Borneo in 2014. The portable rheometer allows, for the first time, natural complex fluids such as pitcher plant trapping fluid to be studied in an ecological context. This will help to answer important questions such as how fluid viscoelasticity influences the trapping success of the plant or how it might depend on the presence of infauna or prey in the fluid. Although the device was developed with pitcher plants in mind, it can be used for studying a wide range of viscous and viscoelastic fluids, including sticky secretions, nectar, honeydew, resins, rubbers, saps, slimes, mucus and other biological fluids (of plant, animal or microbial origin).

We have confirmed Seymour’s capacity to work at 40°C, which makes it suitable for the study of biomedical fluids at physiological temperatures. Virtually all the parts are sterilisable, so it can be used in sterile environments and decontaminated readily. We have not tested the performance at temperatures below 20°C: however, it is reasonable to assume that the lower limit of the operating range is around 0°C as the solenoid will be affected by frost. We expect the device to work well over a broad range of relative humidities but would advise to avoid extremes (*i.e*. working between 20-80%) as excessive evaporation or condensation will affect the sample volume, rendering the measurements invalid.

## Methods

**Figure 11:**
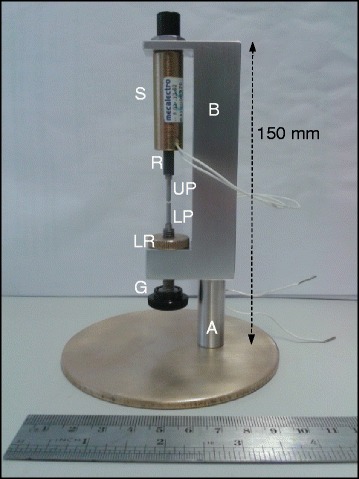
**Photograph of Seymour device.** Labelled parts: S – solenoid; R – end of solenoid rod; UP -upper (moving) piston, LP - lower (stationary) piston used to set the initial gap; LR – locking ring; G – gap adjustment screw; B – Seymour body; A – stand and base.

The labelled photograph in Figure 11 shows the key components of the device. Separation of the platens was provided by a solenoid (electromagnet; model 8.02.13.62, HE & BS Benson Ltd, Newmarket, UK [29]) drawing 4 W power at 6 V DC with a duty cycle rating of 100%. Power to the solenoid was provided by 4×1.5 V AA batteries *via* a switch. The solenoid rod (which provides the upper platen) was replaced with a lighter version with the top end capped to stop it falling through the coil when de-energised. The bottom end was machined from aluminium to give a flat, 1.2 mm diameter platen. The lower platen was similar, but constructed with a screw thread so that its location could be adjusted to set the initial gap and locked in place by a securing knob. The fluid volume was defined by that of a cylinder with initial gap, *g*_0_, and the platen diameter, *r*:

6 $$ V=\pi {r}^2{\mathbf{g}}_o $$

with *r* = 0.6 mm. The distance that the upper platen travelled, *i.e*. the final stretched distance, was adjusted by moving a rubber ‘O’-ring along the shaft. This stopped the rod moving when it contacted the solenoid housing. The platen surfaces were readily wetted by all the liquids tested and filaments always broke by extending (high strain).

The range of operating conditions was established by preliminary tests on *N. maxima* and *Nepenthes* samples from the Cambridge botanical gardens using a Cambridge Trimaster Mk II device [16,30]. The results indicated that an initial gap, *g*_0_, of 0.3-0.7 mm and final stretched distance, *g*_f_, of 1–2 mm was desirable. The Seymour device was constructed with platens of similar diameter to the Trimaster, of 1.2 mm, and maximum final separation of 4 mm in order to accommodate more viscous liquids if needed. These dimensions were sufficiently small to avoid gravitational effects in the cylindrical sample. The influence of gravity is given by the Bond number, *Bo*, defined

7 $$ Bo\equiv \frac{\rho g{L}^2}{\alpha } $$

where *ρ* is the fluid density, *α* the surface tension, *g* the gravitational acceleration and *L* the length scale, here the sample initial diameter, *D*_0_. Bond numbers greater than 1 indicate that gravity effects will be significant. For the aqueous solutions studied here, *Bo* was always less than 0.5.

Key dimensions (e.g. initial and final stretched gap) in movie frames were calculated using the known platen and rod dimensions. The stainless steel body of Seymour was mounted on a (detachable) brass disc for stability. The mass of the assembly, of 350 g, was comparable to that of the camera and lens (720 g). The unit was small enough to fit inside a Stable Micro Systems Total Immersion Temperature Chamber for studies at temperatures and humidities likely to be encountered in tropical testing.

### Image capture and processing

Images of the filaments were captured using a Leica Monozoom 7 lens, illuminated in laboratory experiments by a fibre optic light source. A small battery-powered torchlight mounted on a bracket, subsequently proved to give satisfactory contrast for the Seymour unit. Initial commissioning and comparison studies employed a Photron SA3-60 K-M1-LCA high speed camera operating at 5000 fps with a shutter speed of 10^−5^ s, which gave images with 512×256 pixels. Image acquisition was achieved through the Photron FastCam Viewer software and processed using the TriVision software tool [30] running LabView® on a standard PC. Field trials employed a Ximea MQ003MG-CM (maximum frame rate 500 fps giving images of 648×488 pixels) using the Streampix6® software tool on a laptop for image acquisition. Data processing was performed using (*i*) the TriVision software and (*ii*) a new application scripted for the open source image processing code Fiji, based on code provided by Damian Vadillo (currently at Akzo Nobel Research).

Image processing performed two functions. After the sample was loaded and before platen separation, dimensions in the image capture window were calibrated using the known piston diameter. During separation, extracting the minimum width of the filament requires locating the midpoint. On the Trimaster the gap is set by moving the platens an equal distance from the mid-plane and the operation is straightforward (Figure 1(a)). On the Seymour, the location varies with the gap and so the filament shape has to be identified and its minimum located (Figure 1(b)). The frame rate, illumination and threshold settings required adjustment to ensure that the filament could be identified readily.

The Fiji code automated image analysis for a sequence of frames: it allowed the individual frames to be rotated, if required, to be cropped to remove artefacts that would otherwise hinder the analysis, and it located and measured the filament midpoint in each frame. The output from this code is a text file that can be directly imported into Matlab® or Excel® for further analysis.

The Seymour design has not been patented as the authors consider it to be a logical extension of the existing art. A technical drawing and the Fiji image analysis code can be obtained on request from the authors. Additional file 6: Figure S2 demonstrates the three stages of the image acquisition and analysis, going from the raw images acquired with the high speed camera, to the image masks generated for mid-filament detection to the final location, and value, of the mid-filament diameter.

### Measuring protocol

The platens were initially cleaned with isopropanol and dried with a lens cloth. The illumination and camera settings were checked and trials run to establish the initial and final gap settings. The initial and final gap heights were chosen such that a filament formed and broke after the platens stopped moving. An estimate of these heights could be obtained by slowly lowering the bottom piston using the screw (labelled G on Figure 11) until the filament just broke, and measuring the piston displacement. The piston was then reset to its starting position and the O ring that sets the stroke length for the upper (moving) piston set to the appropriate position using a feeler gauge. Feeler gauges were also used to set the initial gap height.

A micropipette was used to load the test sample so that it fully covered both platens and filled the gap in between. Any excess liquid was carefully removed with a lens tissue. Video recording was started before the solenoid was activated in order to collect frames for calculating dimensions. Video recording was stopped when the filament broke and the image files (saved in .tiff format) were then transferred to the analysis tools. The initial filament diameter, *D*_0_, was taken as the value when the platens stopped moving and time *t* was counted from this event. The pistons were cleaned between experimental sessions using ethanol or isopropanol.

### Note on regression analysis

Fitting data to the Giesekus model, Equation (3), was sensitive to the initial estimates provided to the regression algorithm. The value for the Newtonian viscosity obtained from fitting Equation (1) to the data set provided an upper bound for the viscosity parameter, *η*_0_, so 10% of this value was taken as the initial estimate. Similarly, the relaxation time obtained from fitting the data to Equation (2) was used as the initial estimate of the relaxation time.

### Benchmarking studies

A number of experiments were performed to benchmark the performance of the Seymour device against its archetype, the Trimaster Mk II. Initially, the gap separation was characterised for both devices using identical stretching settings (initial and final gap width). Key parameters such as separation speed, total separation time, piston overshoot and damping were measured for both devices. Subsequently, both devices were used in direct comparison to measure (*i*) a silicone oil (Newtonian liquid), and (*ii*) solutions of carboxymethylcellulose (CMC), a biopolymer exhibiting non-Newtonian behaviour in solution.

The silicone oil (Silicone fluid f191/1300, batch no. 805, Ambersil Ltd, UK) had a viscosity of 2.37 Pa s. Aqueous solutions of 2 wt% carboxymethylcellulose (molecular weight approximately 750 kDa, BDH Chemicals, UK) were prepared by gentle stirring for 4 h. CMC solutions are viscoelastic shear-thinning fluids and are relatively stable [25]. The shear viscosity of the silicone oil and CMC solutions was measured on a Bohlin CVO 120 HR controlled stress rheometer using 25 mm diameter, smooth parallel plates with a 0.5 mm gap at 22°C. The shear rates studied ranged from 0.1 to 3000 s^−1^. The volume of fluid required for these tests (250 μL) was considerably larger than that needed for the Seymour tests (approximately 1 μL). The CMC solution exhibited shear thinning with a zero shear rate viscosity, *η*_0_, of 2.95 Pa s, similar to the viscosity of the silicone oil.

### Botanical garden pitcher plant studies

Samples of pitcher plant fluid for *N. maxima* and *N. alata* were obtained from individual plants at the Cambridge Botanic Gardens, while *N. rafflesiana* and *N. eymae* fluids were obtained from Kew Gardens, London. Greenhouse relative humidity levels were maintained between 34% and 92%. Freshly opened pitchers were chosen where possible. Ideally, fluid would be extracted from an unopened pitcher using a syringe, but in many cases the pitchers had already opened, and there was some contamination by captured flies and plant detritus in the fluid. Approximately 5 ml of liquid was removed and stored in a small sterilised Nalgene bottle. Samples were stored at room temperature (around 22°C), and tested repeatedly over a period of 37 days (*N. eymae*)/16 days (*N. maxima*).

### Field trials

**Figure 12:**
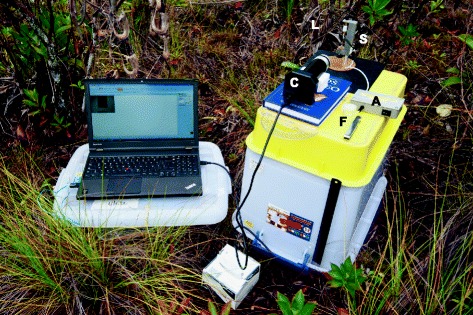
**Seymour device in field testing.** Labels: C – camera; L – lens; S – Seymour extensional rheometer; F – feeler gauges; A – solenoid switch.

Field measurements were performed in Brunei Darussalam, NW Borneo, between July and September 2014. The test fluids used for the benchmarking studies (silicone oil, CMC) were used to calibrate the measurements in the field. Fluid samples (100 μL per sample) were taken from just opening *N. rafflesiana* pitchers using a micropipette and were either measured immediately on site (Figure 12, see also the Additional file 1: video), or transferred to 2 mL sterile screw-top vials and transported back to a nearby field station. Within a maximum of 4 h after sampling, these samples were measured in a room at temperatures between 24°C and 27°C. The pitcher plant fluid samples were then stored at room temperature (22-30°C) and re-measured after 20 days. Storage at room temperature was chosen to mimic conditions during expeditions or at remote field stations where reliable refrigeration may not be available.

### Confirmation of model assumptions

Discussion of the characteristic timescales for CaBER™ experiments can be found in the literature [8,9,31]; these give indications of when filament stretching is the controlling mechanism. The inertial (Rayleigh) time scales for the tests presented here, given by √(*ρD*_0_^3^/8*α*), ranged from 0.02-0.04 ms whereas the viscous times, (given by *η*_0_*D*_0_/2*α*), lay between 2 and 4 ms. The majority of tests were then considerably longer than both these times. The elastocapillary number, comparing the relaxation time in extension to the viscous time, *Ec*= 2*λα*/*η*_0_*D*_0_, ranged from 9 for the greenhouse *N. maxima* tests to 2100 for the wild *N. rafflesiana* (Figure 10). These data confirm the presence of viscoelasticity.

## Additional files

Additional file 1:
**Video Operation of Seymour in the field, measuring a sample of** ***N. rafflesiana*** **pitcher fluid in the natural habitat of the plant in Brunei Darussalam, Borneo.**
Additional file 2: Table S1. Effect of storage at room temperature on the goodness of fit (quantified by the correlation coefficient, *R* ^2^), for *N. eymae* and *N. maxima* fluid obtained from greenhouses (one pitcher for each species). Additional file 3: Figure S1. Effect of storage under chilled conditions. Filament thinning behaviour for greenhouse-sourced *N. maxima* pitcher fluid. Sample tested at Day 1 then stored at 4°C for up to 13 days, for comparison with Figure 7(b). Each aliquot was brought to room temperature before testing. Data decimated for clarity. Additional file 4: Table S2. Fitted model parameters for 11 newly-opened pitchers of *N. rafflesiana* grown in the field. Additional file 5: Table S3.
**Fitted model parameters for 9 of the 11 pitchers of** ***N. rafflesiana*** **in Additional file** 4 **: Table S2 following storage for 20 days at 25-30°C.** Two experimental measurements were made for each pitcher. Samples 13b and 23 were not tested. Additional file 6: Figure S2: Frames from a typical image sequence. The figure shows the original and processed images at the start of a typical filament thinning experiment, two midpoint filament thinning times and at a time close to filament breakup.

## Abbreviations

BOAS: Beads on a string
CMC: Carboxymethylcellulose
UCM: Upper Convected Maxwell (rheological model)
