# Supplementary material for: A portable extensional rheometer for measuring the viscoelasticity of pitcher plant and other sticky liquids in the field
Source: Plant Methods. 2015 Mar 7;11:16. doi: 10.1186/s13007-015-0059-5 (PMC4367843; doi:10.1186/s13007-015-0059-5)
Supplement: Additional file 2: Table S1. — Effect of storage at room temperature on the goodness of fit (quantified by the correlation coefficient, R 2), for N. eymae and N. maxima fluid obtained from greenhouses (one pitcher for each species). [file 13007_2015_59_MOESM2_ESM.doc]

Table S1. Effect of storage at room temperature on the goodness of fit (quantified by the correlation coefficient, *R*^2^), for *N. eymae* and *N. maxima* fluid obtained from greenhouses (one pitcher for each species).

| Species | Storage time (day) | Equation (1) | | Equation (2) | | Equation (3) | | | | |
| --- | --- | --- | --- | --- | --- | --- | --- | --- | --- | --- |
|  |  | *η*_0_  (Pa s) | *R^2^* | *λ*_UCM_  (ms) | *R^2^* | *η*_0_  (Pa s) | *a*  (-) | *λ*_G_  (ms) | | *R^2^* |
| *N. eymae* | 1 | 30.3 | 0.378 | 28.5 | 0.957 | 0.053 | 0 | 31.0 | | 0.976 |
|  | 2 | 16.4 | 0.651 | 15.8 | 0.975 | 0.067 | 0 | 17.0 | | 0.972 |
|  | 7 | 16.3 | 0.717 | 12.9 | 0.997 | 0.032 | 0 | 13.1 | | 0.996 |
|  | 9 | 12.3 | 0.714 | 11.7 | 0.994 | 0.679 | 9.76×10^-4^ | | 12.0 | 0.991 |
|  | 14 | 8.21 | 0.717 | 8.64 | 0.996 | 0 | 0 | 8.64 | | 0.996 |
|  | 23 | 1.72 | 0.844 | 2.21 | 0.998 | 0.004 | 1.43×10^-4^ | 2.54 | | 0.999 |
|  | 37 | 1.41 | 0.996 | 0.73 | 0.950 | 0.435 | 1.30×10^-1^ | 3.46 | | 0.999 |
| *N. maxima* | 1 | 23.6 | 0.355 | 19.6 | 0.991 | 0 | 0 | 20.7 | | 0.994 |
|  | 2 | 14.3 | 0.293 | 11.7 | 0.986 | 0 | 0 | 12.6 | | 0.991 |
|  | 6 | 6.84 | 0.792 | 4.25 | 0.991 | 0.913 | 1.67×10^-2^ | 4.78 | | 0.999 |
|  | 8 | 5.97 | 0.906 | 3.49 | 0.979 | 0.193 | 7.98×10^-3^ | 6.25 | | 0.998 |
|  | 16 | 3.14 | 0.929 | 1.08 | 0.989 | 0.0016 | 1.03×10^-4^ | 1.74 | | 0.995 |
