# Supplementary figures and images for: A portable extensional rheometer for measuring the viscoelasticity of pitcher plant and other sticky liquids in the field
Source: Plant Methods. 2015 Mar 7;11:16. doi: 10.1186/s13007-015-0059-5 (PMC4367843; doi:10.1186/s13007-015-0059-5)

Figure S1


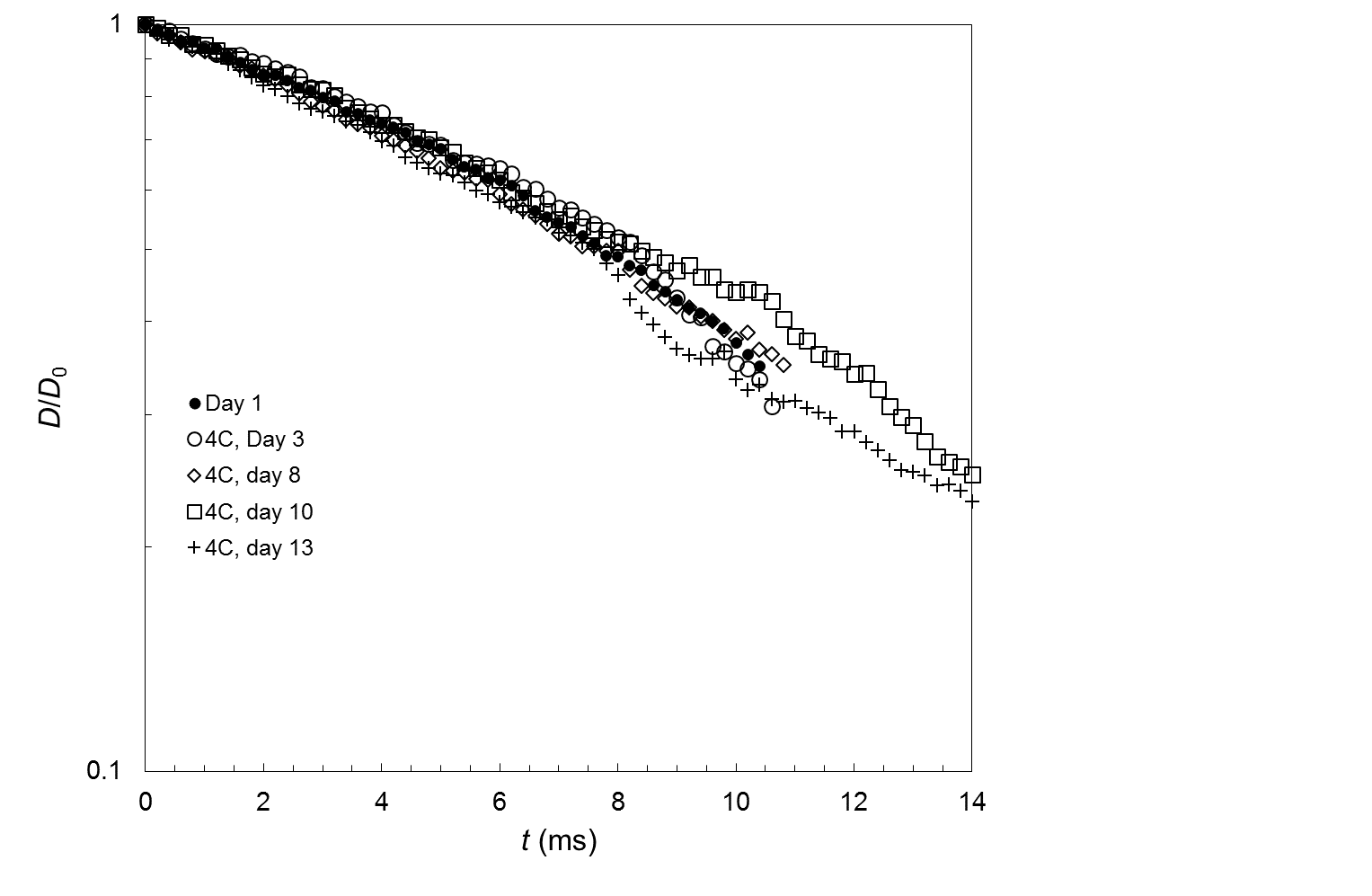

Supplement: Additional file 3: Figure S1. — Effect of storage under chilled conditions. Filament thinning behaviour for greenhouse-sourced N. maxima pitcher fluid. Sample tested at Day 1 then stored at 4°C for up to 13 days, for comparison with Figure 7(b). Each aliquot was brought to room temperature before testing. Data decimated for clarity. [file 13007_2015_59_MOESM3_ESM.docx]

## Slide 1
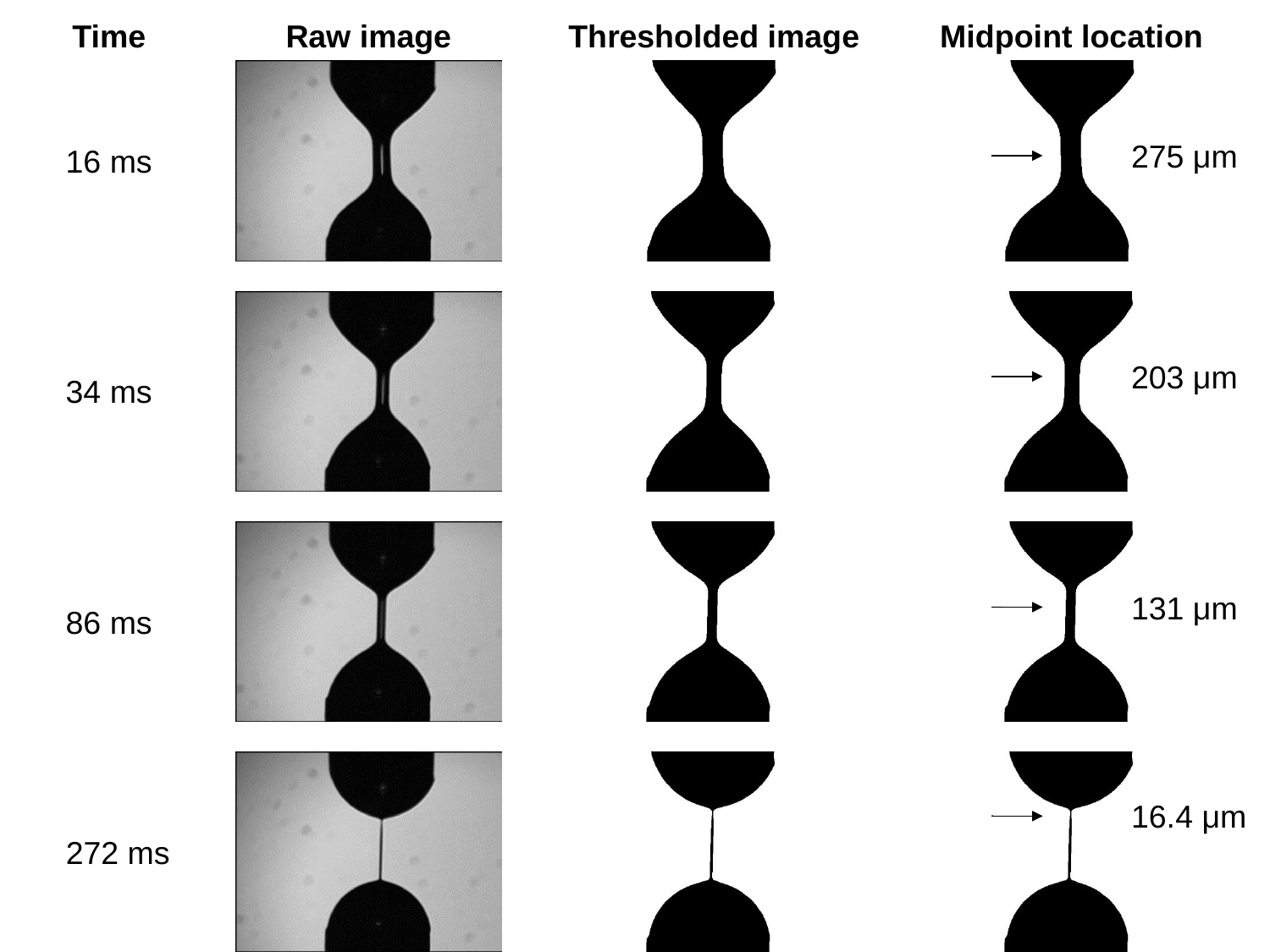

Time
16 ms
34 ms
86 ms
272 ms
Raw image
Thresholded image
Midpoint location
275 μm
203 μm
131 μm
16.4 μm
1.2 mm

Supplement: Additional file 6: Figure S2: — Frames from a typical image sequence. The figure shows the original and processed images at the start of a typical filament thinning experiment, two midpoint filament thinning times and at a time close to filament breakup. [file 13007_2015_59_MOESM6_ESM.pptx]
