# Supplementary material for: A portable extensional rheometer for measuring the viscoelasticity of pitcher plant and other sticky liquids in the field
Source: Plant Methods. 2015 Mar 7;11:16. doi: 10.1186/s13007-015-0059-5 (PMC4367843; doi:10.1186/s13007-015-0059-5)
Supplement: Additional file 4: Table S2. — Fitted model parameters for 11 newly-opened pitchers of N. rafflesiana grown in the field. [file 13007_2015_59_MOESM4_ESM.doc]

Table S2. Fitted model parameters for 11 newly-opened pitchers of *N. rafflesiana* grown in the field.

| Pitcher number | Equation (1) | | Equation (2) | | Equation (3) | | | |
| --- | --- | --- | --- | --- | --- | --- | --- | --- |
|  | *η*_0_  (Pa s) | *R^2^* | *λ*_UCM_  (ms) | *R^2^* | *η*_0_  (Pa s) | *a*  (-) | *λ*_G_  (ms) | *R^2^* |
| 13 | 191 | 0.531 | 515 | 0.979 | 12.2 | 2.0×10^-3^ | 610 | 0.986 |
| 13b | 39.9 | 0.770 | 76.3 | 0.982 | 4.40 | 1.0×10^-2^ | 130 | 0.997 |
| 15 | 44.1 | 0.788 | 79.0 | 0.971 | 4.50 | 2.0×10^-3^ | 85.0 | 0.996 |
| 16 | 243 | 0.823 | 894 | 0.992 | 45.0 | 1.0×10^-3^ | 880 | 0.993 |
| 17 | 229 | 0.823 | 583 | 0.989 | 37.0 | 5.0×10^-3^ | 600 | 0.993 |
| 18 | 229 | 0.681 | 434 | 0.990 | 27.0 | 4.5×10^-3^ | 470 | 0.995 |
| 19 | 221 | 0.916 | 558 | 0.984 | 36.0 | 4.5×10^-3^ | 620 | 0.989 |
| 20 | 318 | 0.609 | 1210 | 0.997 | 40.0 | 7.0×10^-4^ | 1210 | 0.997 |
| 21 | 100 | 0.661 | 221 | 0.992 | 21.0 | 4.0×10^-3^ | 230 | 0.996 |
| 22 | 94.6 | 0.632 | 217 | 0.984 | 8.10 | 2.5×10^-3^ | 265 | 0.991 |
| 23 | 185 | 0.770 | 963 | 0.947 | 42.5 | 1.0×10^-3^ | 960 | 0.958 |
