# Supplementary material for: A portable extensional rheometer for measuring the viscoelasticity of pitcher plant and other sticky liquids in the field
Source: Plant Methods. 2015 Mar 7;11:16. doi: 10.1186/s13007-015-0059-5 (PMC4367843; doi:10.1186/s13007-015-0059-5)
Supplement: Additional file 5: Table S3. — Fitted model parameters for 9 of the 11 pitchers of N. rafflesiana in Additional file 4 : Table S2 following storage for 20 days at 25-30°C. Two experimental measurements were made for each pitcher. Samples 13b and 23 were not tested. [file 13007_2015_59_MOESM5_ESM.doc]

Table S3. Fitted model parameters for 9 of the 11 pitchers of *N. rafflesiana* in Table S2 following storage for 20 days at 25-30°C. Two experimental measurements were made for each pitcher. Samples 13b and 23 were not tested.

| Pitcher number | Equation (1) | | Equation (2) | | Equation (3) | | | |
| --- | --- | --- | --- | --- | --- | --- | --- | --- |
|  | *η*_0_  (Pa s) | *R^2^* | *λ*_UCM_  (ms) | *R^2^* | *η*_0_  (Pa s) | *a*  (-) | *λ*_G_  (ms) | *R^2^* |
| 13 | 256 | 0.733 | 729 | 0.988 | 28.0 | 4.0×10^-3^ | 820 | 0.997 |
|  | 245 | 0.865 | 485 | 0.996 | 21.0 | 6.0×10^-3^ | 660 | 0.999 |
| 15 | 37.4 | 0.964 | 79.1 | 0.995 | 3.16 | 5.6×10^-3^ | 105 | 0.997 |
|  | 30.1 | 0.931 | 69.0 | 0.994 | 3.90 | 4.8×10^-3^ | 87.0 | 0.994 |
| 16 | 8.52 | 0.881 | 25.4 | 0.995 | 0.650 | 2.1×10^-3^ | 30.2 | 0.998 |
|  | 7.84 | 0.886 | 23.1 | 0.995 | 0.656 | 2.2×10^-3^ | 27.0 | 0.998 |
| 17 | 134 | 0.887 | 350 | 0.990 | 22.2 | 5.7×10^-3^ | 405 | 0.995 |
|  | 122 | 0.824 | 326 | 0.983 | 22.5 | 5.7×10^-3^ | 380 | 0.994 |
| 18 | 104 | 0.864 | 259 | 0.986 | 4.20 | 1.1×10^-3^ | 308 | 0.996 |
|  | 681 | 0.870 | 169 | 0.989 | 4.00 | 0.9×10^-3^ | 180 | 0.997 |
| 19 | 104 | 0.839 | 284 | 0.982 | 20.5 | 9.0×10^-3^ | 360 | 0.991 |
|  | 122 | 0.897 | 343 | 0.991 | 29.2 | 8.4×10^-3^ | 390 | 0.996 |
| 20 | 244 | 0.748 | 502 | 0.988 | 22.0 | 4.0×10^-3^ | 640 | 0.993 |
|  | 242 | 0.708 | 519 | 0.987 | 39.0 | 9.0×10^-3^ | 650 | 0.993 |
| 21 | 4.08 | 0.894 | 11.5 | 0.997 | 0.160 | 1.6×10^-3^ | 14.3 | 0.997 |
|  | 3.50 | 0.886 | 10.3 | 0.997 | 0.130 | 1.0×10^-3^ | 12.4 | 0.998 |
| 22 | 30.0 | 0.843 | 79.2 | 0.986 | 2.70 | 2.4×10^-3^ | 93.5 | 0.995 |
|  | 38.8 | 0.929 | 96.9 | 0.994 | 2.85 | 3.1×10^-3^ | 123 | 0.997 |
